# Within-System Agreement Between Real-Time and Post-Processed Data Using Dynamix from League Optical Tracking (Hawk-Eye) in Professional Football

**DOI:** 10.3390/sports14050202

**Published:** 2026-05-15

**Authors:** Marco Beato, Paolo Troiani, Chiara Zinco, Dario Pompa, Maurizio Bertollo, Cristian Savoia

**Affiliations:** 1 Department of Wellbeing, Nutrition and Sport, Pegaso Open University, 80143 Naples, Italy; marcobeato.coach@gmail.com; 2 Department of Medicine and Aging Sciences, University “G. d’Annunzio” of Chieti-Pescara, 66100 Chieti, Italy; paolo.troiani@outlook.com (P.T.); m.bertollo@unich.it (M.B.); 3 Sport Data Analyst Program, ITS Leonardo Academy Foundation, 24127 Bergamo, Italy; chiarazinco@gmail.com; 4 BIND-Behavioral Imaging and Neural Dynamics Center, University “G. d’Annunzio” of Chieti-Pescara, 66100 Chieti, Italy; 5 The Research Institute for Sport and Exercise Sciences, The Tom Reilly Building, Liverpool John Moores University, Liverpool L3 5AH, UK; cristiansavoia@gmail.com

**Keywords:** soccer, tracking, monitoring, team sport, kinematic

## Abstract

This study aimed to evaluate the within-system agreement and interchangeability of real-time and post-processed external load metrics in elite football. Data were collected from 50 official Serie A matches using Dynamix (K-Sport World S.R.L., Pesaro, Italy), the platform for acquiring and standardizing tracking inputs. SmartLive, a real-time monitoring module embedded within Dynamix, was compared with post-processed data from the league-approved optical tracking provider (Hawk-Eye Innovations Limited, Basingstoke, UK) in Serie A. The external load metrics analyzed included total distance covered; distances at speeds exceeding 15, 20, and 25 km·h^−1^; distances within the 15–20 km·h^−1^ and 20–25 km·h^−1^ ranges; distance covered during accelerations > 2 m·s^−2^ and decelerations < −2 m·s^−2^; and peak speed. Intraclass correlation coefficients (ICCs) demonstrated excellent agreement across all metrics, with values ranging from 0.929 to 0.999. Bland–Altman analysis revealed small mean differences between systems, indicating strong agreement. Overall, the findings confirm that both real-time and post-processed data are in close agreement across a wide range of performance metrics. Minor discrepancies were observed in intermediate speed zones and acceleration/deceleration events. This study provides the first validation of SmartLive’s within-system agreement with post-processed data, supporting its use alongside post-processed data in elite football environments.

## 1. Introduction

Football (soccer), as one of the most globally followed sports, continues to evolve through its integration of advanced technology and data-driven methodologies [1,2]. At the forefront of this evolution, Serie A, the Italian premier professional football league, is recognized for its high competitiveness, tactical and physical performance [3,4], and it is one of the main European football championships (together with Spanish, German and English premier leagues). Clubs within this league operate within highly developed performance infrastructures, relying on advanced technology to extract meaningful insights from matches and training sessions [5,6]. Such technology can be used for decision-making processes by coaches, performance analysts, and sports scientists [2,6]. Consequently, the accuracy and reliability of the data informing these decisions are paramount [7].

To address this need, player tracking technologies have been commonly implemented in elite football clubs for the monitoring of physical performance [8]. These systems provide critical insights into external load metrics, enabling practitioners to assess physical output, optimize training loads, and mitigate injury risks [9,10]. Previous research has demonstrated that tracking systems can accurately monitor a wide range of performance metrics, including average accelerations and decelerations [8,11]. However, each validated system operates independently from others due to differences in data processing algorithms and technological specifications [12]. As a result, validation findings from one model or brand cannot be directly transferred to another. Currently, player tracking in Serie A relies on league-approved optical providers such as Hawk-Eye (Hawk-Eye Innovations Limited, Basingstoke, UK) [13]. Such data are post-processed using the Dynamix platform (K-Sport World S.R.L., Pesaro, Italy), designed for detailed post-match analysis and long-term trend identification [13] (i.e., data analyzed after the match using more advanced filtering procedures). Within this framework, SmartLive (a real-time monitoring module embedded in the Dynamix platform) processes the same optical data stream to deliver immediate positional and movement information, allowing for tactical evaluation and in-game adjustments (i.e., data processed and available during the match). The methodological differences between these analysis modules, particularly in processing protocols (e.g., different filters are used because of the different time available for analysis), introduce variables that may influence the comparability and consistency of the metrics they produce [10,14]. This was previously reported for other inter-system comparisons, spanning from video tracking systems to Global Positioning System (GPS) [14,15,16]. From a practical standpoint, real-time outputs inform in-game decisions such as substitutions, fatigue alerts, and intensity monitoring, whereas post-processed outputs inform post-match debriefs and longitudinal monitoring; any systematic discrepancy between the two could therefore lead to inconsistent decisions across these complementary workflows.

As clubs and governing bodies (i.e., Fédération Internationale de Football Association [FIFA]) increasingly integrate these systems interchangeably or in parallel, ensuring inter-system reliability becomes a critical concern [14,17]. Reliable and consistent data across different technologies allows for seamless performance comparisons, effective longitudinal tracking, and coherent integration into centralized databases used for league-wide analysis [18]. Torres-Ronda et al. [14], in their review, stated that when choosing among the wide range of metrics offered by these systems and interpreting time series data, it is important to critically assess both their accuracy (in terms of validity and reliability) and their relevance to real-world scenarios. Specifically, high levels of agreement between systems (i.e., between-system reliability) are essential for preserving the integrity of performance assessments. An example of this is by Taberner et al. [19], who assessed the between-system agreement (and interchangeability) of optical tracking technologies, specifically, eight cameras sampling at 10 Hz (PROZONE^®^) and six high-definition motion cameras sampling at 25 Hz (TRACAB^®^). The study concluded that there was potential overestimation of the sprint running load demands in the English Premier League, although total distance and high-speed running were considered interchangeable [19].

The current study responds to the need for a robust evaluation of agreement between real-time and post-processed data. Because both data streams are generated from the same Hawk-Eye optical source and differ exclusively in the processing pipeline applied, the present study addresses within-system agreement rather than a comparison between independent tracking technologies. Utilizing an extensive dataset drawn from 50 official Serie A matches, the research provides a comprehensive comparison of external load metrics—including total distance covered, high-speed running, accelerations, and peak speed measurements, which are all common metrics monitored in football [20,21]. This is the first study that has investigated the within-system agreement and interchangeability of real-time vs. post-processed data generated from the same optical tracking source. Therefore, the aim is to determine the extent of agreement between the two approaches and to assess their interchangeability in elite football environments. By investigating the alignment of these data sources, the study contributes to the ongoing discourse around performance monitoring standardization in professional football. The findings are poised to inform practitioners, technology providers, and governing bodies about best practices for integrating tracking systems and maintaining reliable performance data streams across competitions.

## 2. Materials and Methods

### 2.1. Participants

The inclusion criteria required players to have participated in official Serie A matches during the season, with no restrictions on total match time. Goalkeepers were excluded from the analysis to maintain consistency in positional demands; therefore, only match data from outfield players were included. Although the study was based on a convenience sample of professional Serie A players (*n* = 221) [22], this yielded more than 1000 paired observations for total distance and for most of the metrics analyzed. Post hoc considerations indicate that, for a Bland–Altman agreement analysis, only a few dozen to at most ~100 paired observations would typically be required to estimate limits of agreement with a 95% confidence interval half-width of ±100 m, depending on the SD of the paired differences. Likewise, using a conventional framework (α = 0.05, power = 0.80), approximately 200 paired observations would be sufficient to detect a small systematic bias (effect size = 0.2). Therefore, the actual sample size in this study substantially exceeds the minimum requirements for both precision of the limits of agreement and power to detect small between-method differences. The dataset was obtained via tracking systems routinely used by K-Sport for performance monitoring. Player data were collected as part of contractual obligations during competitive matches throughout the season. As the monitoring formed part of the company’s ongoing operational practices, formal ethical approval from an institutional review board was not deemed necessary [23]. Nonetheless, all personal and team-specific data were anonymized prior to analysis. The study was conducted in accordance with the ethical principles outlined in the Declaration of Helsinki, thereby safeguarding the confidentiality and welfare of all participating individuals.

### 2.2. Tracking Systems

This study analyzed external load metrics of 50 official Serie A matches collected via Hawk-Eye (Hawk-Eye Innovations Limited, Basingstoke, UK). Hawk-Eye’s workflows typically include triangulation-based position estimation, video calibration, and delayed filtering, aimed at maximizing post-match accuracy [13]. Tracking data were analyzed by Dynamix (K-Sport World S.R.L., Pesaro, Italy), software V1.25.6, which is the central platform used to acquire and standardize tracking inputs [4,6]. SmartLive (software, V19F) is a real-time monitoring module embedded within Dynamix. It functions as an integrated feature within the broader performance ecosystem offered by K-Sport, allowing for continuous acquisition, visualization, and analysis of player tracking data during live match-play. As a software and cloud-based application, SmartLive supports decision-making by providing real-time performance metrics and customizable visual alerts (software version 2025). The comparison between real-time and post-processed data across several key external load metrics, including total distance covered, distances covered at speeds greater than 15, 20, and 25 km·h^−1^, as well as distances within the 15–20 km·h^−1^ and 20–25 km·h^−1^ ranges [7]. Additionally, we examined the distance covered during accelerations > 2 m·s^−2^, decelerations < −2 m·s^−2^, and the peak speed (km·h^−1^) recorded during each match [24]. Both real-time (SmartLive) and post-processed (Dynamix) outputs analyzed in this study were generated from the same Hawk-Eye optical feed; therefore, any difference between the two data streams arises exclusively from the processing pipeline applied and not from the use of different tracking systems.

### 2.3. Real-Time Data Analysis

SmartLive utilizes video tracking data captured from the league’s official optical tracking provider (in the present study, exclusively Hawk-Eye [18]) and processes these inputs in real time within the Dynamix software environment [4,6]. The system applies customized filtering and smoothing algorithms adapted for live optical tracking conditions, ensuring that data latency is minimized for in-match usability. Details regarding the customized filtering and smoothing algorithms cannot be disclosed, as they constitute confidential intellectual property. Real-time outputs are visualized through multiple display formats, including 2D field animations, player-specific battery indicators, and color-coded alert systems. These features enable staff to monitor performance thresholds and high-intensity efforts (e.g., >100% of historical maximum intensity minute) and to receive alerts when athletes approach their cumulative physical limits. Notably, all the metrics evaluated in this study are monitored in real time by SmartLive, offering practitioners immediate insights into player training load and fatigue. To contextualize the comparative analysis and the rationale behind the statistical tests employed, it is important to note the differences in processing workflows between real-time and post-processed data. Both workflows are managed within the Dynamix platform: SmartLive, as a dedicated module, processes the Hawk-Eye optical feed in real time to deliver immediate feedback through live transmission and on-field visualization, whereas the standard Dynamix pipeline generates post-processed outputs optimized for detailed post-match analysis.

### 2.4. Statistical Analysis

Normality of the data was tested using the Shapiro–Wilk test. Statistical significance was set at *p* < 0.05, and 95% confidence intervals were reported. In the present study, agreement refers to the extent to which two parallel outputs (real-time and post-processed) generated from the same optical source produce equivalent values for a given metric; because both outputs are derived from the same matches without repeated measurements over time, the design assesses within-system agreement rather than test–retest reliability. In this manuscript, “agreement” refers to the statistical concordance between the two outputs (quantified through ICC and Bland–Altman analysis), whereas “interchangeability” is used in its applied sense, indicating the extent to which the two outputs can be used in place of one another for practical performance-monitoring decisions. To evaluate agreement, we employed the Intraclass Correlation Coefficient (ICC) using a two-way mixed model [25]. Unlike simple correlation coefficients such as Pearson’s r, which primarily assess the strength and direction of a linear relationship between two variables, the ICC provides a more rigorous measure by evaluating the degree of agreement between parallel measurements [25]. The ICC values were interpreted based on established thresholds, where values > 0.90 were considered *excellent*, values between 0.80 and 0.90 were deemed *good*, 0.70 to 0.80 *acceptable*, 0.60 to 0.70 *questionable*, 0.50 to 0.60 poor, and values < 0.50 *unacceptable* [26]. In addition to ICC, Bland–Altman plots were used to visually assess the absolute agreement (i.e., systematic bias and limits of agreement) between the two systems [27]. These plots report the mean difference and 95% limits of agreement, providing insight into any systematic bias and the range within which most differences between the systems fell. Statistical analysis was performed using JASP software (version 0.19.1, Amsterdam, The Netherlands).

## 3. Results

Descriptive analysis of real-time and post-processed data for the parameter analyzed in this study can be found in Table 1.

The within-system agreement analysis demonstrated excellent agreement across all measured parameters: total distance (ICC = 0.999); distance covered at speeds greater than 15 km·h^−1^ (ICC = 0.997); greater than 20 km·h^−1^ (ICC = 0.998); and greater than 25 km·h^−1^ (ICC = 0.987). For the intermediate speed zones, the ICC for distance covered between 15 and 20 km·h^−1^ was 0.994, and between 20 and 25 km·h^−1^ was 0.995. The reliability for distance covered during accelerations greater than 2 m·s^−2^ and decelerations less than −2 m·s^−2^ was 0.960 for both. Peak speed, while still showing excellent reliability, had a slightly lower ICC of 0.929 (see Table 2).

The Bland–Altman analysis revealed the following mean differences and 95% limits of agreement (±1.96 SD) between real-time and post-processed data: for total distance, the mean difference was 50.4 m with limits of 206.9 and −106.0 m; for distance > 15 km·h^−1^, the mean difference was 48.2 m (38.2; −134.6); for distance > 20 km·h^−1^, 4.1 m (46.1; −37.9); and for distance > 25 km·h^−1^, 12.9 m (42.6; −16.8). The distance covered between 15 and 20 km·h^−1^ showed a mean difference of −52.1 m (26.0; −130.2), while the 20–25 km·h^−1^ range had a mean difference of −8.8 m (33.9; −51.5). For accelerations > 2 m·s^−2^, the mean difference was −43.3 m (32.6; −119.3), and for decelerations < −2 m·s^−2^, it was 19.8 m (111.3; −71.7). The mean difference in peak speed was −0.3 km·h^−1^ with limits of 1.1 and −1.8 km·h^−1^.

Visual inspection of the Bland–Altman plots, which included 95% confidence intervals, confirmed that the agreement of the measurements across the two systems can be found in Figure 1, Figure 2, Figure 3, Figure 4, Figure 5, Figure 6, Figure 7, Figure 8 and Figure 9.

Graphical dependent group analysis for each analyzed metric is reported in the Supplementary Materials (Figures S1–S9).

## 4. Discussion

This study aims to evaluate the within-system agreement and interchangeability of real-time and post-processed external load metrics in elite football. The within-system agreement (real-time and post-processed data) was found to be excellent across all measured parameters (range ICC = 0.960–0.999). It should be acknowledged, however, that ICC values of this magnitude are partly expected given that both outputs are derived from the same Hawk-Eye optical feed and differ only in the processing pipeline applied; consequently, in this design, the Bland–Altman analysis carries greater interpretative weight than the ICCs alone. However, the slightly lower agreement observed for peak speed (ICC = 0.929), while still within the excellent range, suggests that practitioners requiring high precision for this specific metric should interpret the data with caution or consider applying system-specific calibration. To further explore the agreement and potential bias between the two systems, we used some Bland–Altman plots, which found some small differences between the two data processing systems.

ICC provides a rigorous measure by evaluating the degree of agreement between parallel measurements [25]; therefore, ICC is particularly suitable for assessing the agreement between different processing pipelines that claim to quantify the same construct—especially when applied to the dynamic and high-performance setting of elite football [14]. In this study, the comparison between real-time and post-processed data revealed high within-system agreement across a comprehensive array of external load parameters. Such an agreement suggests that the data derived from these systems are practically applicable for performance monitoring, decision-making, and longitudinal analyses in professional football environments such as Serie A. Specifically, the ICC values presented indicate excellent agreement for several metrics. For example, total distance covered demonstrated an ICC of 0.999, reflecting virtually complete alignment in outputs between systems. Similarly, metrics for running over different speed thresholds, whether defined as >15 km·h^−1^ (0.997), >20 km·h^−1^ (0.998), or >25 km·h^−1^ (0.987), show strong agreement. Even within different speed zones—such as 15–20 km·h^−1^ (0.994) and 20–25 km·h^−1^ (0.995)—the systems produced highly reliable measurements. A previous study that compared TRACAB^®^ and PROZONE^®^ found that total distance displayed a perfect (r = 0.99) correlation, while high-speed running and sprinting distance displayed very large (r = 0.81 and r = 0.73) correlations [19]. While total distance is nearly perfectly correlated between the systems, for high-speed zones, these tracking systems exhibited substantial differences compared to those analyzed in the present study (see Table 2). Differences in filtering algorithms, data resolution, or signal processing techniques (between systems) could contribute to subtle discrepancies in how such movements are detected and measured [18,19].

Regarding acceleration and deceleration actions, previous research suggests that these more transient, high-frequency movement patterns might present greater challenges for consistent quantification, as previously demonstrated for other tracking technologies [12,28]. However, this study found some slightly lower yet still excellent ICC values (>2 m·s^−2^ and <−2 m·s^−2^, both 0.960). The high ICCs support both systems’ utility for monitoring mechanical demands and high-intensity efforts—a crucial aspect of elite performance [29,30]. Finally, peak speed, often regarded as a key metric for evaluating top-end sprinting performance, showed an ICC of 0.929—still within the range of excellent reliability, though marginally lower than other parameters. The analysis of peak speed was consistent with findings from other inter-model technologies, although some discrepancies between studies still persist [31,32,33]. For instance, the inter-model reliability of previously validated GPS (i.e., STATSports Apex vs. Viper) ranged between ICC = 0.92–0.98 depending on the sprint length analyzed [34]. The authors of this study think that this small deviation (between real-time and post-processed reliability for peak speed data) may be due to inherent differences in data filtering and processing; however, further research is needed to verify this. Taken together, these findings (ICC analysis reported in Table 2) demonstrate that both real-time and post-processed analyses provide robust and consistent data outputs across a range of performance indicators, although the strength of the agreement should be confirmed by inspection of the Bland–Altman analysis. However, the authors emphasize that a margin of error still exists.

**Figure 1:**
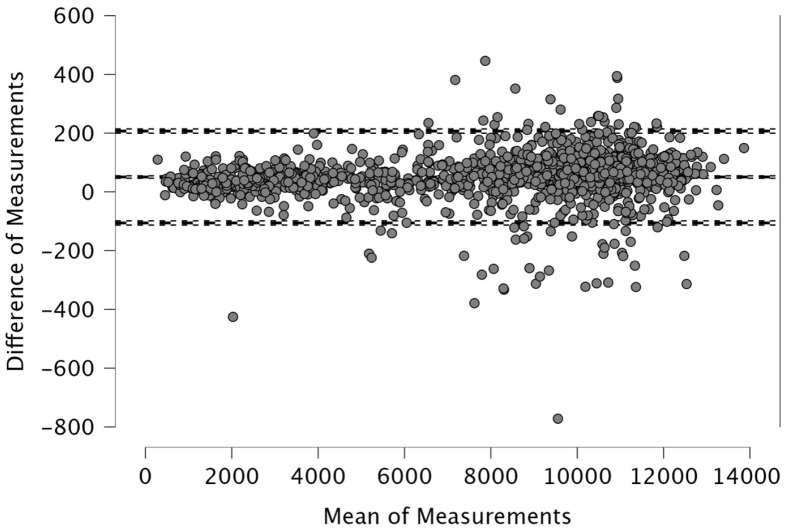
Total distance, real-time and post-processed data (*n* = 1424 data points). The Bland–Altman analysis revealed the following mean differences and 95% limits of agreement.

**Figure 2:**
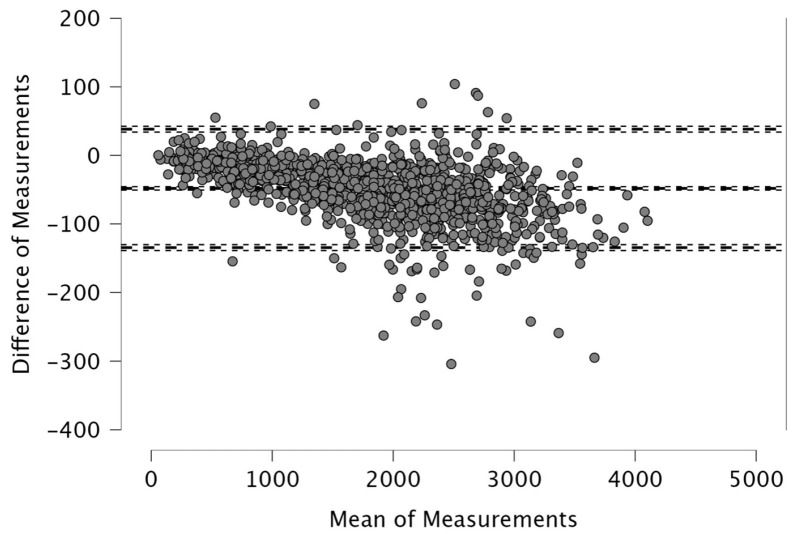
Distance covered >15 km·h^−1^. Real-time and post-processed data (*n* = 1224 data points). The Bland–Altman analysis revealed the following mean differences and 95% limits of agreement.

**Figure 3:**
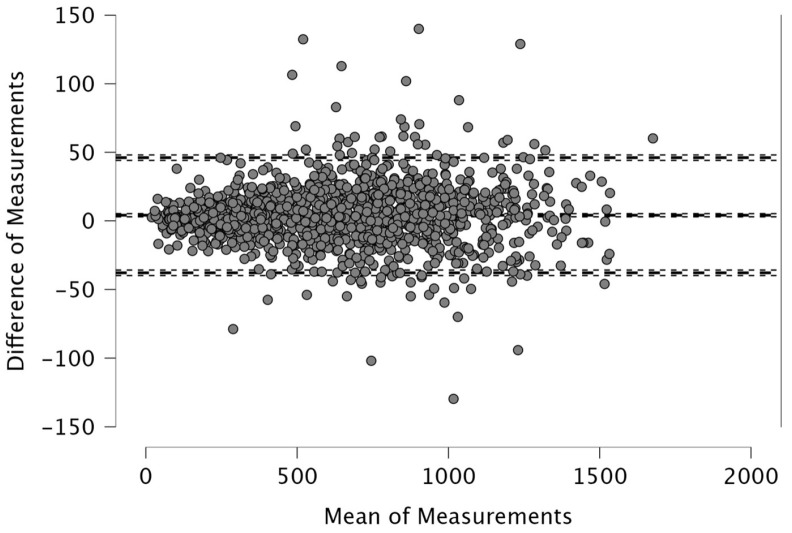
Distance covered >20 km·h^−1^. Real-time and post-processed data (*n* = 1335 data points). The Bland–Altman analysis revealed the following mean differences and 95% limits of agreement.

**Figure 4:**
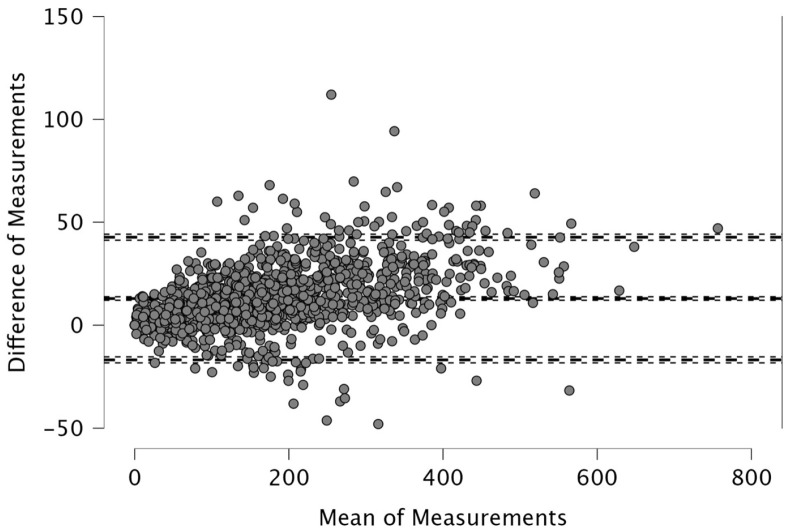
Distance covered >25 km·h^−1^. Real-time and post-processed data (*n* = 1248 data points). The Bland–Altman analysis revealed the following mean differences and 95% limits of agreement.

**Figure 5:**
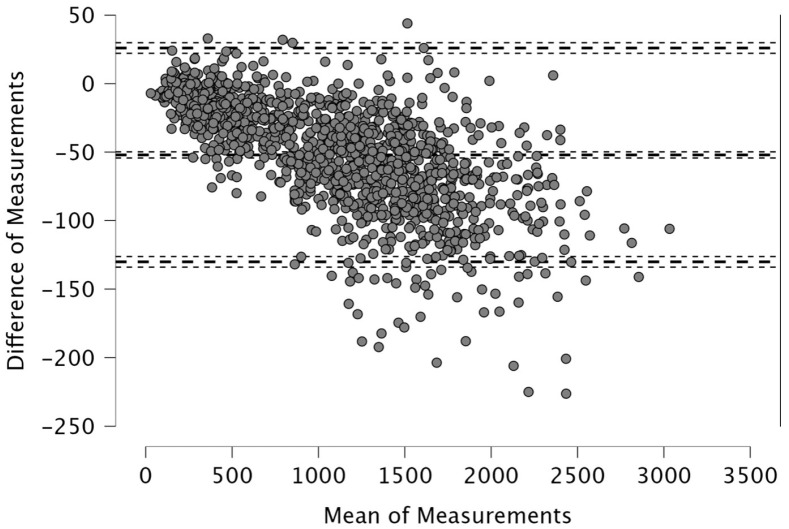
Distance covered between 15 and 20 km·h^−1^. Real-time and post-processed data (*n* = 1224 data points). The Bland–Altman analysis revealed the following mean differences and 95% limits of agreement.

**Figure 6:**
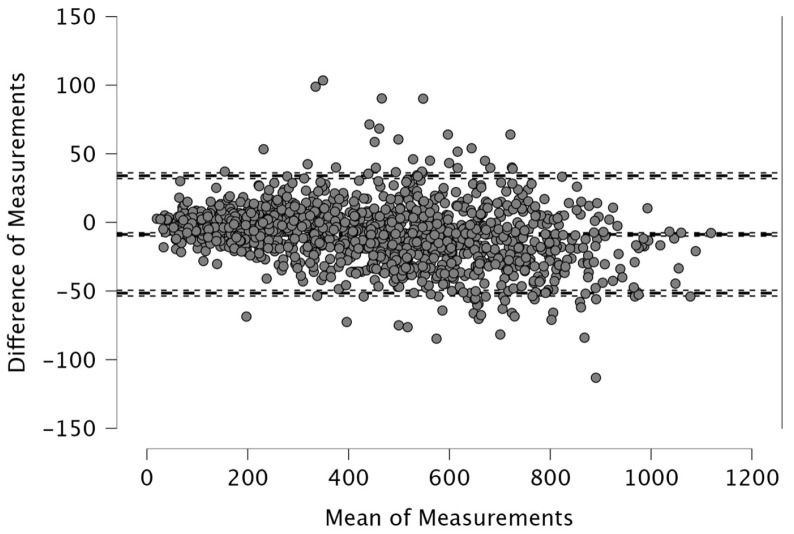
Distance covered between 20 and 25 km·h^−1^. Real-time and post-processed data (*n* = 1248 data points). The Bland–Altman analysis revealed the following mean differences and 95% limits of agreement.

The present study employed Bland–Altman analysis to assess the level of agreement between the two data analysis processes, real-time and post-processed, across a range of external load parameters collected during official Serie A matches. This method enabled a direct comparison of measurement outputs and facilitated the identification of systematic bias and random variation between the systems [35]. The results revealed that the mean differences between them were generally small and fell within acceptable limits of agreement. For total distance, the average discrepancy was 50.4 m, with bounds ranging from −106.0 to 206.9 m (Figure 1). This finding, while showing a modest bias, suggests strong concordance, especially considering the cumulative nature of this metric across entire match durations. Regarding total distance, Taberner et al. [19] found a 484.16 m (5%) difference between PROZONE^®^ and TRACAB^®^ systems. Another study compared an optical tracking system (Mediacoach System) and a GPS device (Wimu Pro) and found that the distance error was 103.8 m (2.27%) [36]. Both studies reported greater inter-model error than what was found in the current study (50.4 m, 0.6%). It is evident that technological advancements have significantly enhanced measurement accuracy over time. For example, Randers et al. (2010) reported substantial discrepancies between systems—specifically, a semi-automatic multiple-camera system, a video-based time-motion analysis system, and two GPS models operating at acquisition frequencies of 1 Hz and 5 Hz—in the quantification of absolute distances covered [37], which is different from what has been found in this study and in the most recent scientific literature. Similar to the high agreement for total distance found in the current study, high agreement was observed in other high-speed running metrics between real-time and post-processed data (Figure 2, Figure 3 and Figure 4). Distances covered at thresholds exceeding 15 km·h^−1^, 20 km·h^−1^, and 25 km·h^−1^ showed mean differences of 48.2 m, 4.1 m, and 12.9 m, respectively. These variances are small within the context of professional match play and would likely not impact performance evaluations or tactical decisions. Previous research reported that comparison of optical tracking (i.e., PROZONE^®^) to GPS has shown slight-to-moderate and moderate-to-large differences for total distance and distance above 18 km∙h^−1^, respectively [38], which are greater than what was found in this study (e.g., Figure 1, Figure 3 and Figure 4). Taberner et al. found that high-speed running (>19.8 km∙h^−1^) and sprinting distance (>25.2 km∙h^−1^) showed differences between systems of −6.58 m (3%) and 98.9 m (61%), respectively, which demonstrates low intermodal reliability for sprinting distances. Moreover, in a very recent study, a comparison between GPS and semi-automated optical tracking systems (Second Spectrum) in English Premier League players revealed consistent differences, though smaller than previously reported [39]. The optical system tended to record higher values for total distance (+4%), high-speed running (+12%), and sprinting (+18%). These results indicate that while discrepancies still exist, especially at higher speeds, advancements in technology have improved the accuracy and alignment between the two systems, making them more dependable for performance analysis [39]. In the current study, we found a mean difference in the distance > 20 km·h^−1^ of 4.1 m (0.6%) and for distance > 25 km·h^−1^ of 12.9 m (7.1%) between real-time and post-processed data. Regarding intermediate speed zones (15–20 km·h^−1^ and 20–25 km·h^−1^), the mean differences were slightly more pronounced at −52.1 m and −8.8 m, respectively (Figure 5 and Figure 6). Although these represent small systematic offsets, the limits of agreement remained narrow enough to support the notion of operational interchangeability. In support of these results, previous research found that sprinting distance showed larger errors than total distance among several tracking devices (during between-model comparison) [18].

**Figure 7:**
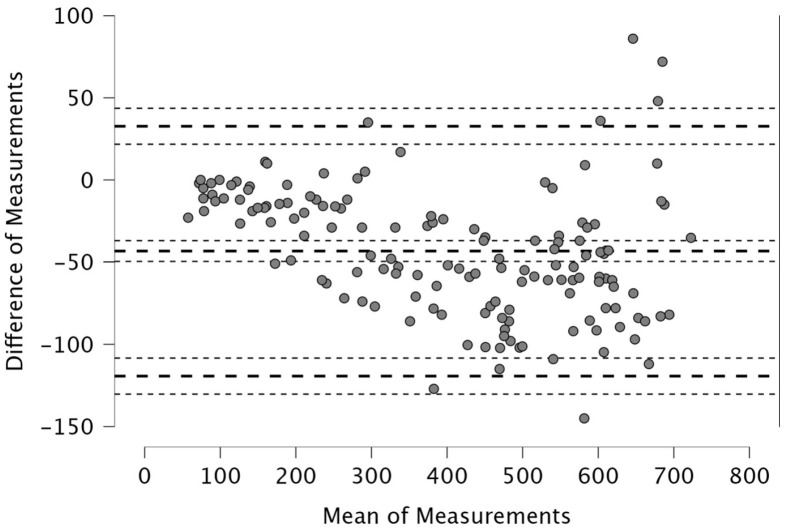
Distance covered during accelerations > 2 m·s^−2^. Real-time and post-processed data (*n* = 147 data points). The Bland–Altman analysis revealed the following mean differences and 95% limits of agreement.

**Figure 8:**
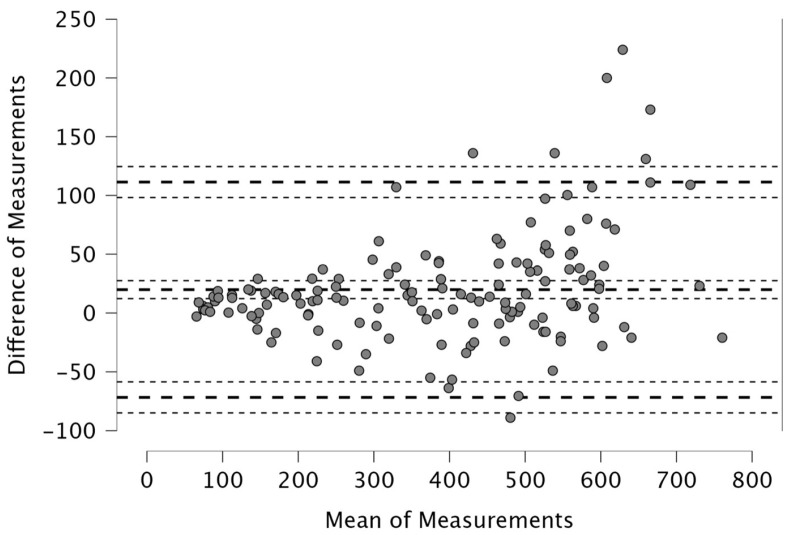
Distance covered during decelerations < −2 m·s^−2^. Real-time and post-processed data (*n* = 147 data points). The Bland–Altman analysis revealed the following mean differences and 95% limits of agreement.

**Figure 9:**
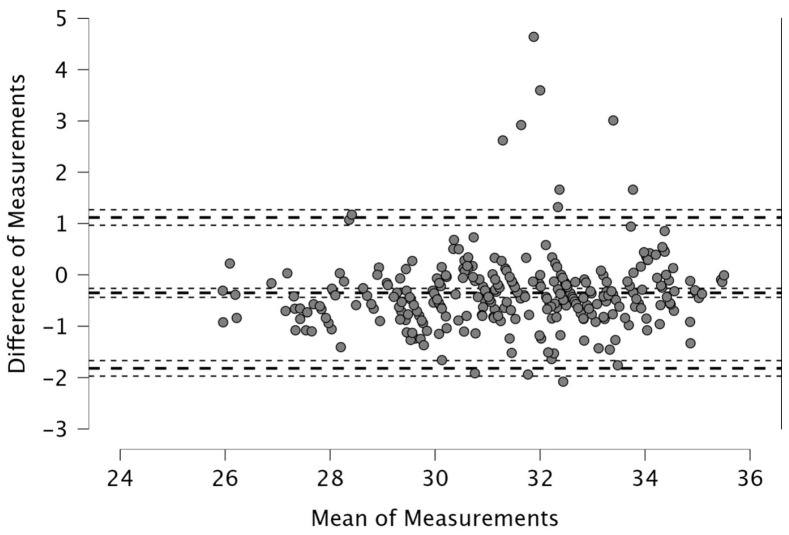
Peak speed (km·h^−1^). Real-time and post-processed data (*n* = 287 data points). The Bland–Altman analysis revealed the following mean differences and 95% limits of agreement.

Movement data associated with accelerative and decelerative efforts also demonstrated good alignment. For accelerations > 2 m·s^−2^, the mean difference was −43.3 m, while decelerations < −2 m·s^−2^ exhibited a difference of 19.8 m (Figure 7 and Figure 8). These deviations may be explained by differences in how each system processes rapid changes in velocity—particularly relevant in moments of high-intensity accelerations, braking and changes in directions [11], where sensor sensitivity and sampling rate can influence recorded output. There is a limited number of studies comparing acceleration and deceleration distances across video tracking systems. In contrast, more extensive data are available from inter-unit reliability analyses in GPS technology [16,40]. Therefore, further research is warranted in this specific area. Regarding peak speed, this metric showed the smallest absolute bias, with a mean difference (from Bland–Altman analysis) of just −0.3 km·h^−1^ (< 1% error) and limits of agreement between −1.8 and 1.1 km·h^−1^ (Figure 9)—this is in line with the 1% error found in between-model reliability analyses in GPS [34] as well as with Pons et al., who found a 0.39 km·h^−1^ difference between Mediacoach System and Wimu Pro [36]. This finding indicates that both real-time and post-processed peak speed data are highly precise, despite their differing data processing. Taken together, the Bland–Altman analyses indicate that the agreement between real-time and post-processed outputs is metric-dependent. Agreement is well supported for total distance and peak speed, where bias and limits of agreement are narrow relative to the magnitude of the metric. Greater relative variability was observed for high-speed running metrics, including both cumulative thresholds (>15, >20, >25 km·h^−1^) and discrete intermediate zones (15–20 km·h^−1^ and 20–25 km·h^−1^). The largest variability was found for acceleration and deceleration distances. For these metrics, the wider limits of agreement suggest that the two outputs should be used interchangeably with caution. Practitioners should preferably rely on a single processing pipeline when acceleration, deceleration, or high-speed running metrics drive performance-monitoring decisions.

### Limitations and Future Directions

As football continues to evolve through increasingly granular performance metrics and high-stakes decision-making, the reliability of tracking technologies remains pivotal to optimizing player management, tactical planning, injury prevention, and performance analysis [14,17]. This study, which analyzed data from 50 competitive matches in Italy’s Serie A during the 2024/25 season, contributes to the growing body of evidence supporting the robustness of current tracking systems. However, a key limitation is the exclusive focus on a single league; therefore, future studies should replicate this analysis using data from other competitions to confirm the generalizability of the findings. Further research should also investigate inter-system agreement under varied match conditions, such as differences in pitch dimensions, weather conditions, and player density, to ensure consistency and accuracy across diverse contexts. Additionally, assessing reliability across a broader spectrum of physiological and technical parameters could enhance the utility of tracking data in multidisciplinary performance environments. Calibration studies aimed at identifying system-specific biases or limitations may also prove valuable, offering insights that could refine existing methodologies and inform the development of next-generation tracking innovations.

## 5. Conclusions

The findings from this study demonstrate that both real-time and post-processed data provide robust and consistent between-model outputs across a diverse range of performance metrics. Despite minor discrepancies observed in certain parameters, such as intermediate speed zones and acceleration/deceleration events, the overall agreement between processing systems is high, and these differences are unlikely to impact the accuracy of performance assessments in practical settings. By confirming the agreement of the two data processing systems, the results offer valuable implications for practitioners in elite football environments, who rely on precise and reliable data for player monitoring, tactical analysis, and longitudinal tracking. The ability to integrate outputs from either system into centralized databases also facilitates standardization across teams and competitions, supporting broader initiatives for data harmonization in sports science.

## Figures and Tables

**Table 1:** Descriptive analysis of real-time and post-processed data.

Metric	Mean Real-Time	SD Real-Time	Mean Post-Processed	SD Post-Processed
Total distance (m)	7630.9	3611.6	7580.4	3598.0
Distance > 15 km·h^−1^ (m)	1731.5	854.8	1791.4	887.0
Distance > 20 km·h^-−1^ (m)	636.1	332.6	638.0	336.5
Distance > 25 km·h^−1^ (m)	181.5	121.3	173.4	120.8
Distance 15–20 km·h^−1^ (m)	1098.9	567.8	1153	596.8
Distance 20–25 km·h^−1^ (m)	451.2	231.3	464.6	239.5
Accelerations > 2 m·s^−2^ (m)	383.0	181.9	434.9	203.1
Decelerations < −2 m·s^−2^ (m)	395.6	187.8	376.6	178.4
Peak speed (km·h^−1^)	31.22	2.18	31.19	2.15

SD = standard deviation.

**Table 2:** ICC analysis of real-time and post-processed data.

Metric	Point Estimate	Lower 95% CI	Upper 95% CI
Total distance	0.999	0.999	1.000
Distance > 15 km·h^−1^	0.997	0.970	0.999
Distance > 20 km·h^−1^	0.998	0.998	0.998
Distance > 25 km·h^−1^	0.987	0.937	0.995
Distance 15–20 km·h^−1^	0.994	0.883	0.998
Distance 20–25 km·h^−1^	0.995	0.992	0.997
Accelerations > 2 m·s^−2^	0.960	0.650	0.986
Decelerations < −2 m·s^−2^	0.960	0.938	0.973
Peak speed (km·h^−1^)	0.929	0.888	0.952

Note: ICC values are based on the Shrout & Fleiss [28] definition—ICC (2,1).

## Data Availability

The data are available from the corresponding authors upon reasonable request. Due to confidentiality agreements with the professional football clubs involved, the datasets cannot be made publicly available.

## Supplementary Materials

The following supporting information can be downloaded at: https://www.mdpi.com/article/10.3390/sports14050202/s1, Figure S1–S9.
