# Supplementary material for: Within-System Agreement Between Real-Time and Post-Processed Data Using Dynamix from League Optical Tracking (Hawk-Eye) in Professional Football
Source: Sports (Basel). 2026 May 15;14(5):202. doi: 10.3390/sports14050202 (PMC13211446; doi:10.3390/sports14050202)

Supplementary analyses

Graphical dependent group analysis

Figure S1. Total distance (TD). Real-time and post-processed data

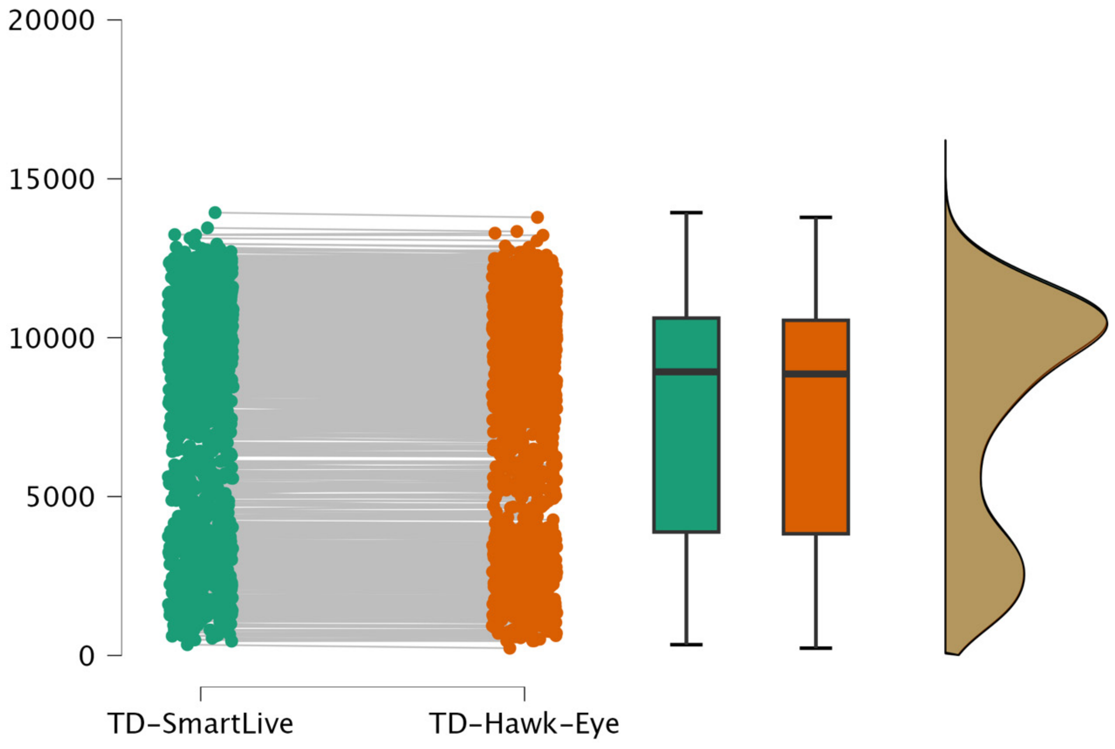

Figure S2. Distance covered > 15 km·h^{-1}. Real-time and post-processed data

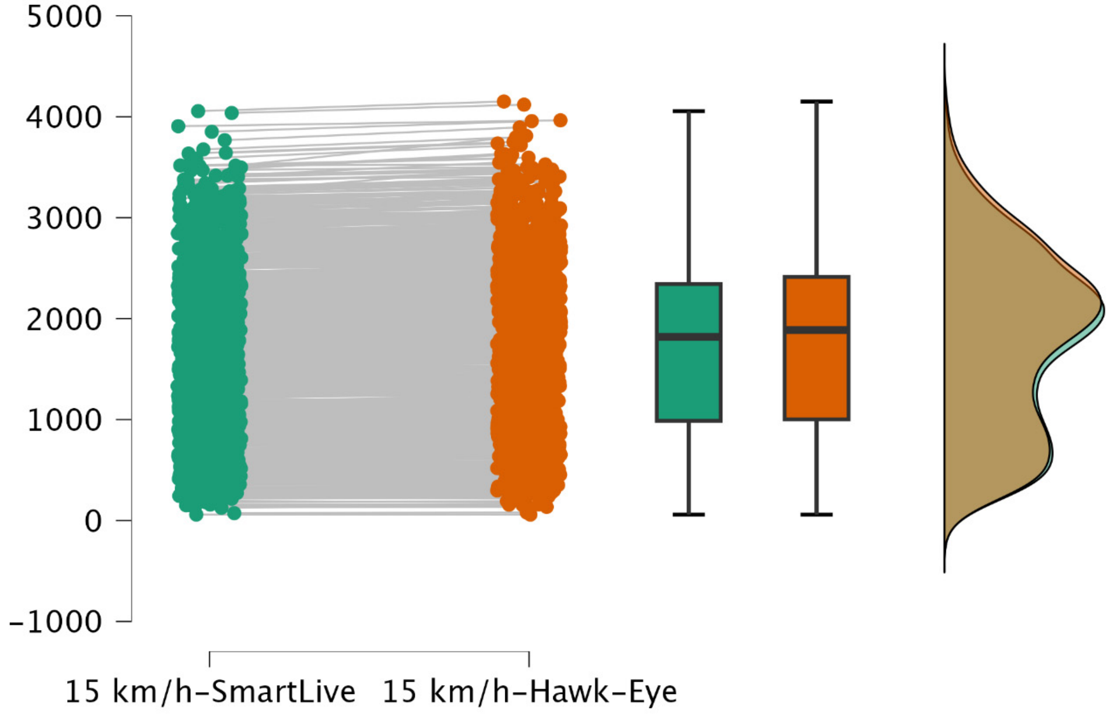

Figure S3. Distance covered > 20 km·h^{-1}. Real-time and post-processed data

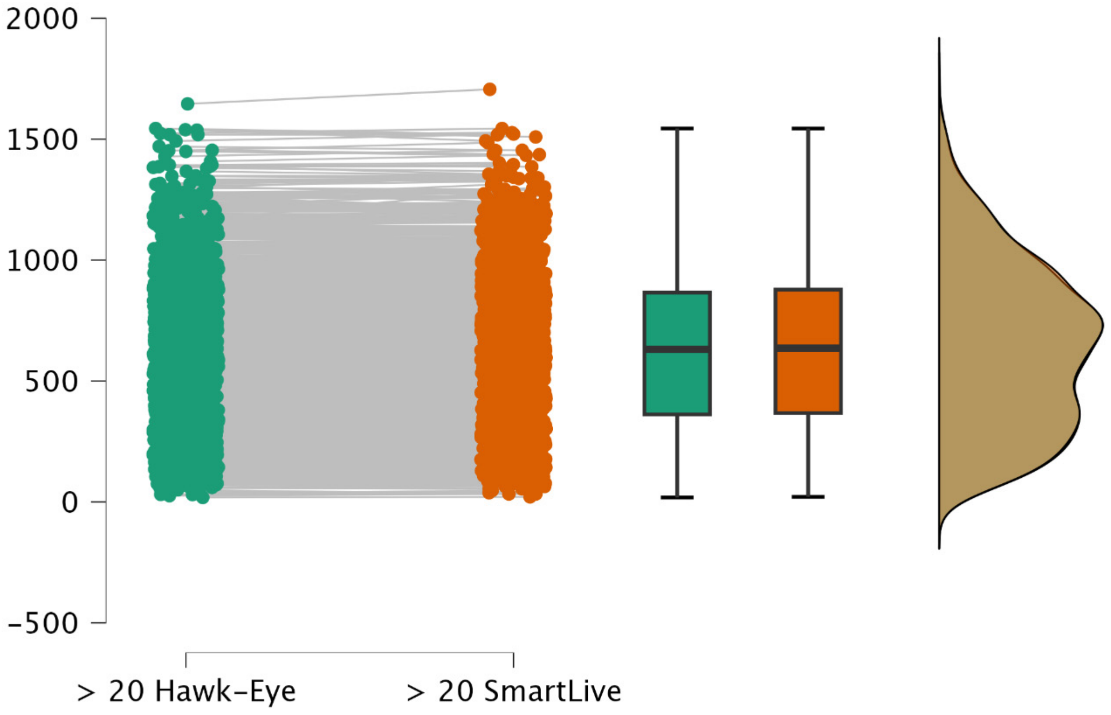

Figure S4. Distance covered > 25 km·h^{-1}. Real-time and post-processed data

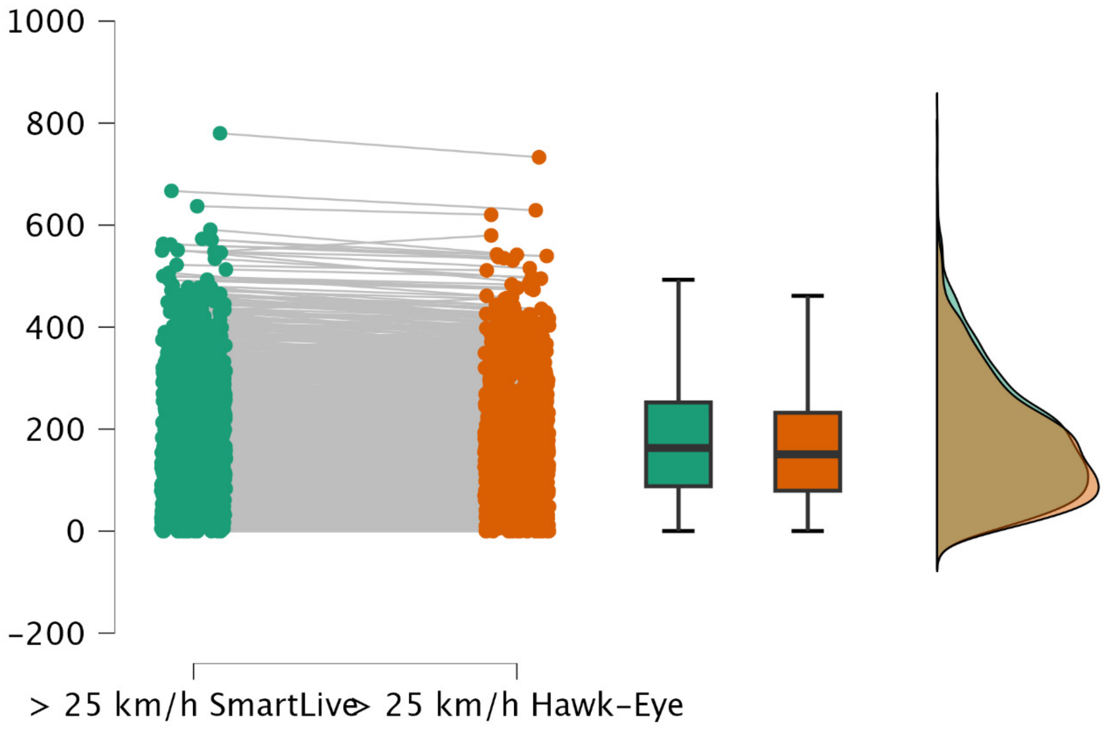

Figure S5. Distance covered between 15-20 km·h^{-1}. Real-time and post-processed data

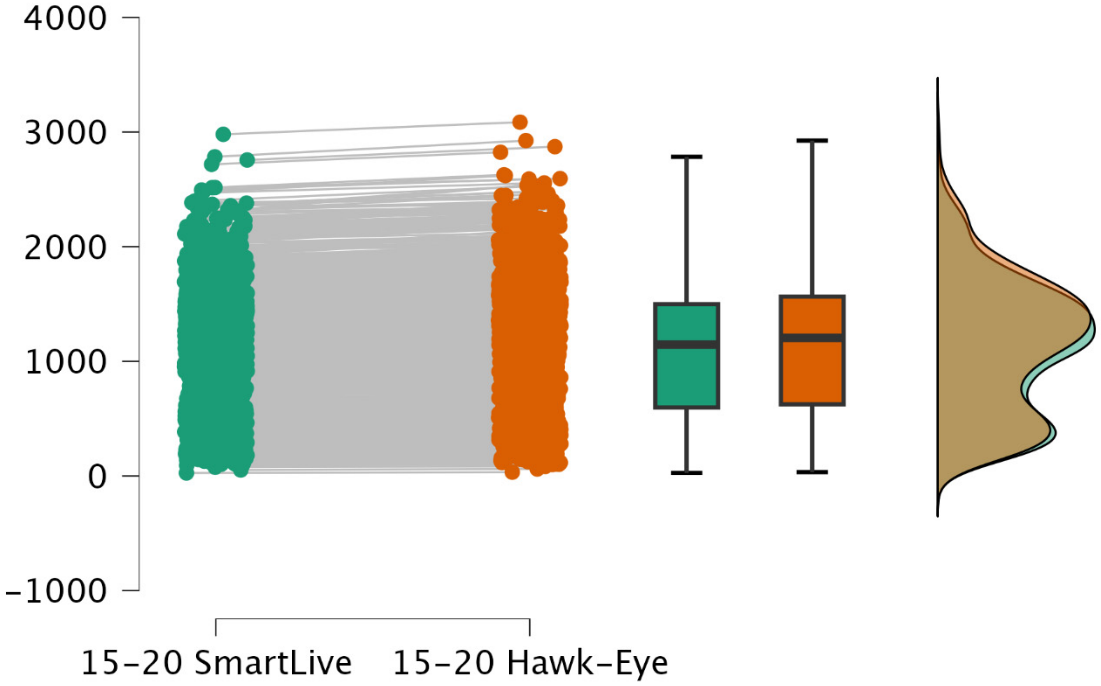

Figure S6. Distance covered between 20-25 km·h^{-1}. Real-time and post-processed data

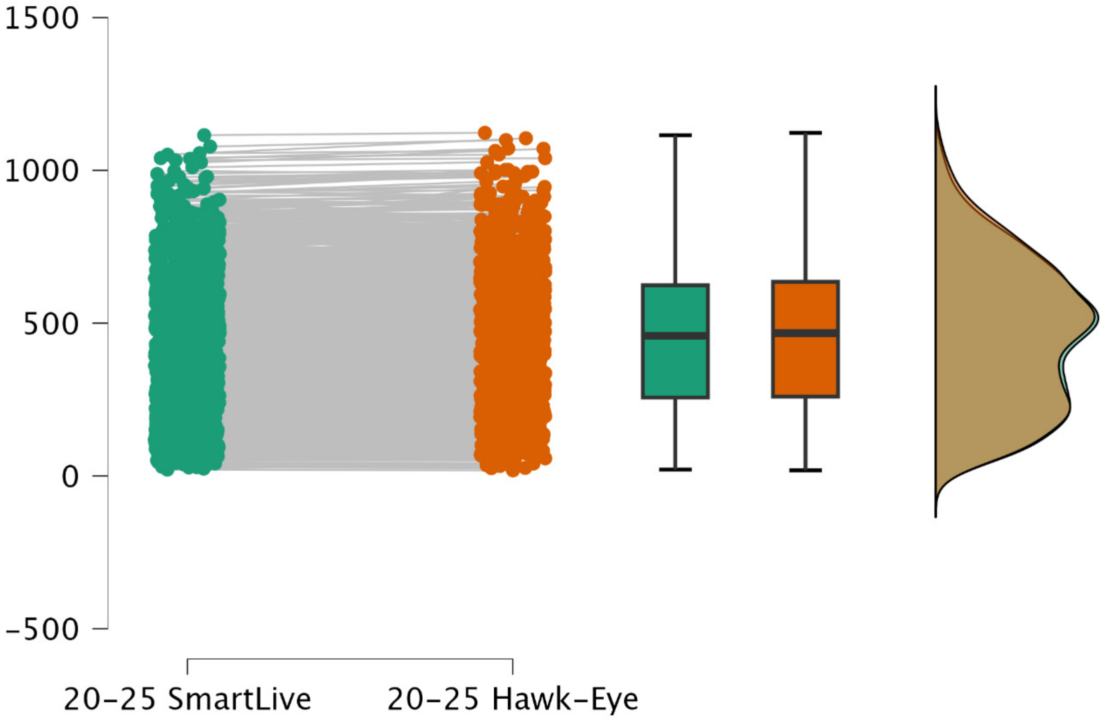

Figure S7. Distance cover during accelerations > 2m·s⁻². Real-time and post-processed data

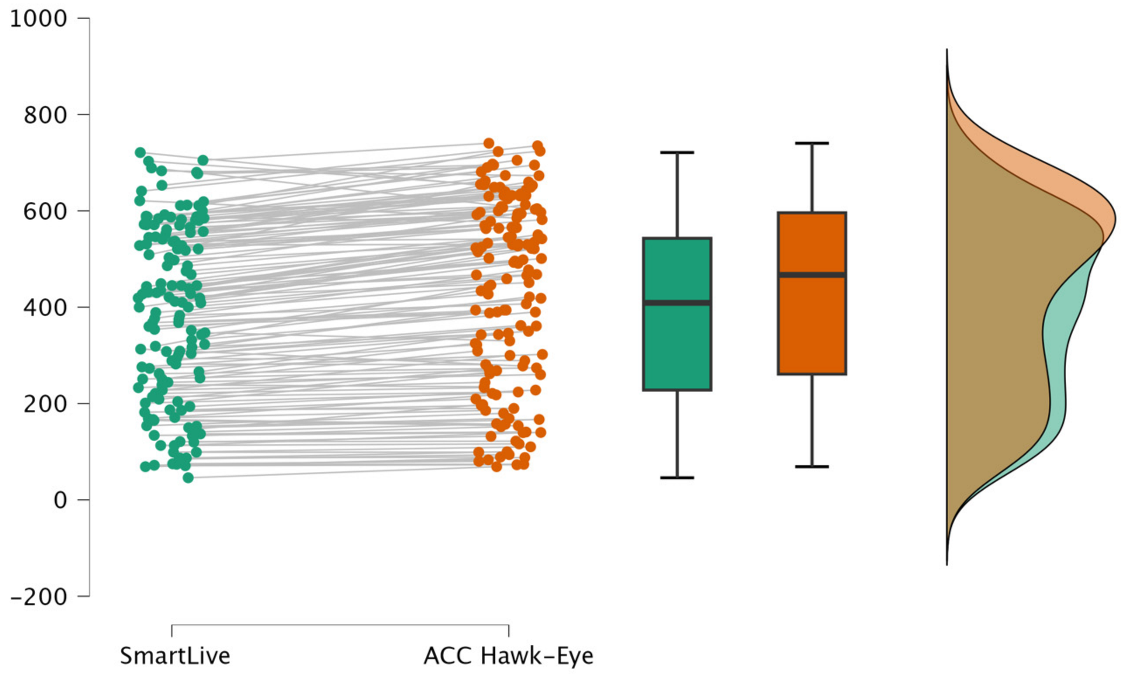

Figure S8. Distance cover during decelerations < -2m·s⁻². Distance. Real-time and post-processed data

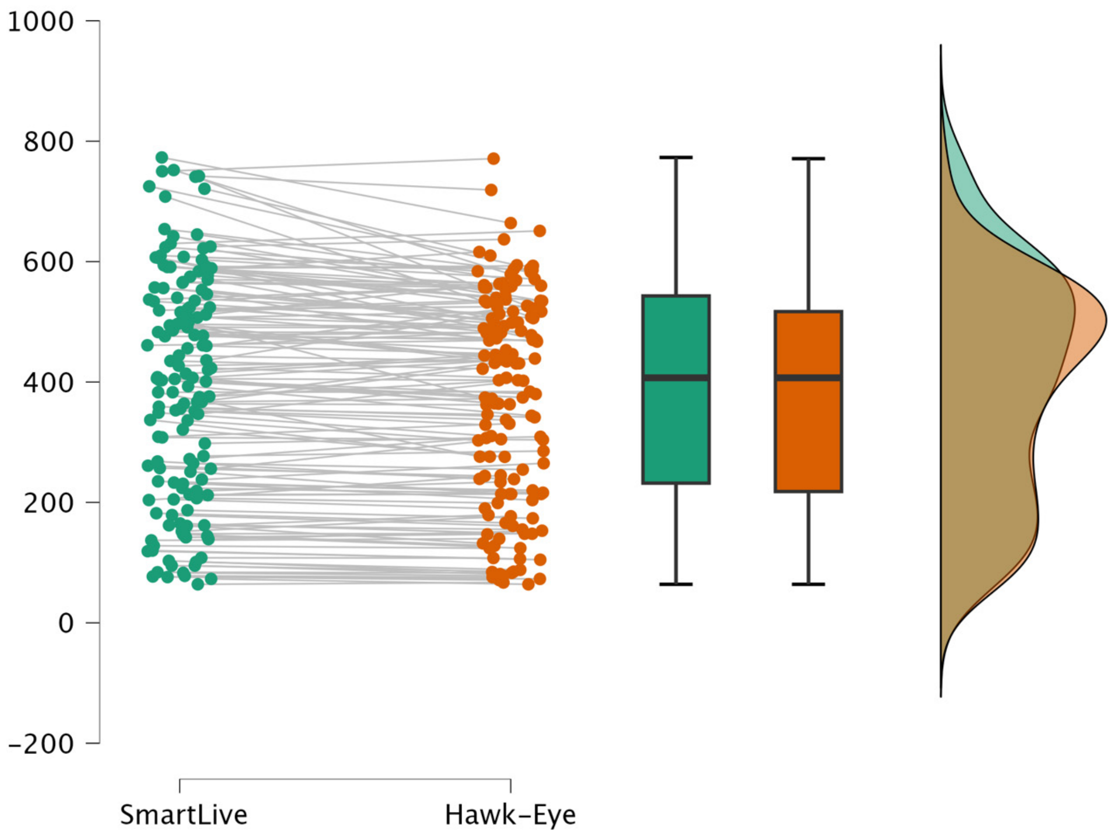

Figure S9. Peak speed (km·h^{-1}) Real-time and post-processed data

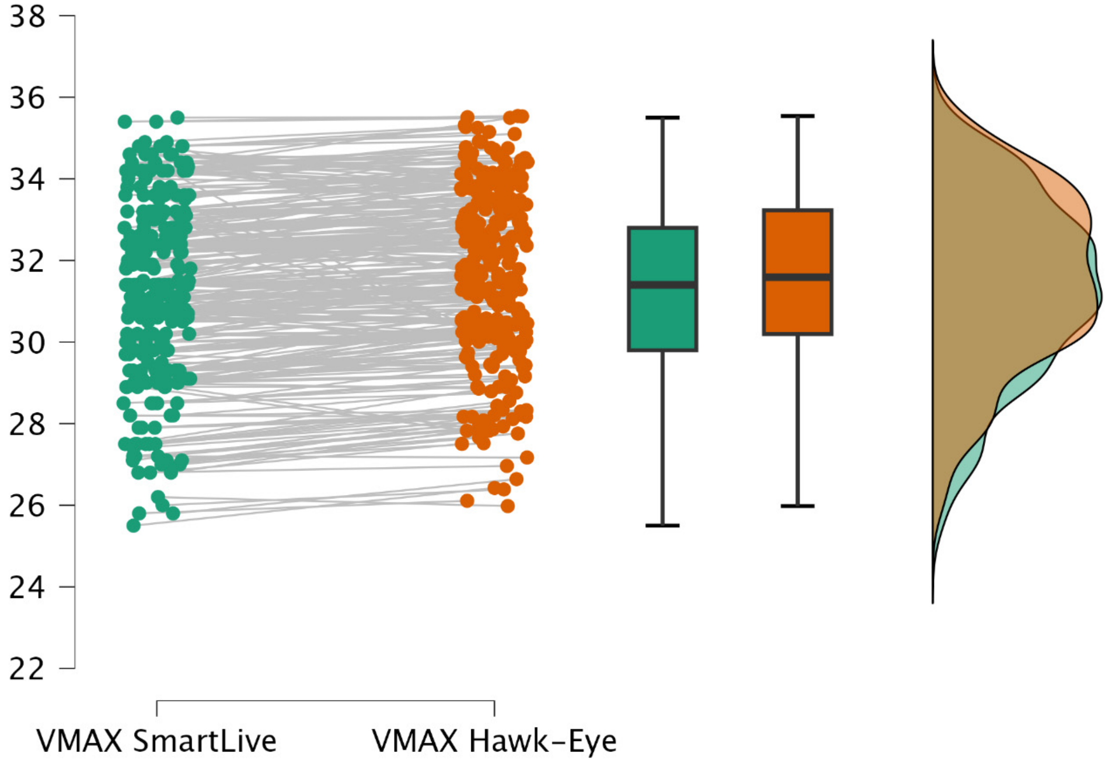

Supplement: Supplementary file 1 [file sports-14-00202-s001.zip › sports-4257849-supplementary.pdf]
